# Getting under the skin of Polycomb-dependent gene regulation

**DOI:** 10.1101/gad.348257.121

**Published:** 2021-03-01

**Authors:** Neil P. Blackledge, Robert J. Klose

**Affiliations:** Department of Biochemistry, University of Oxford, Oxford OX1 3QU, United Kingdom

**Keywords:** Polycomb, PRC1, PRC2, skin, epidermis, stem cell, epigenetics, H2AK119ub, H3K27me3

## Abstract

The Polycomb repressive system functions through chromatin to regulate gene expression and development. In this issue of *Genes & Development*, Cohen and colleagues (pp. 354–366) use the developing mouse epidermis as a model system to show that the two central Polycomb repressive complexes, PRC1 and PRC2, have autonomous yet overlapping functions in repressing Polycomb target genes. They show that this cooperation enables the stable repression of nonepidermal transcription factors that would otherwise compromise epidermal cell identity and disrupt normal skin development.

## Abstract (precis)

This Outlook discusses Cohen et al.’s finding where they use the developing mouse epidermis as a model system to show that the two central Polycomb repressive complexes, PRC1 and PRC2, have autonomous yet overlapping functions in repressing Polycomb target genes.

The Polycomb repressive system post-translationally modifies histones in chromatin to repress transcription and is essential for controlling gene expression during development. This relies on Polycomb repressive complex 1 (PRC1), which monoubiquitylates histone H2A, and Polycomb repressive complex 2 (PRC2), which methylates histone H3 at lysine 27 (Schuettengruber et al. 2017). Biochemical studies have shown that the activities of PRC1 and PRC2 are coupled through their capacity to recognize each other's histone modifications, enabling the convergence of these complexes on a shared set of target gene promoters (Fig. 1A, top panel) (Blackledge et al. 2015). Based on this, it has been widely proposed that the feedback mechanisms that link PRC1 and PRC2 may define how the Polycomb system represses gene expression. However, in the context of development, where Polycomb system function is critical, it remains unclear to what extent coupling between PRC1 and PRC2 underpins Polycomb-dependent gene regulation.

**Figure 1.**
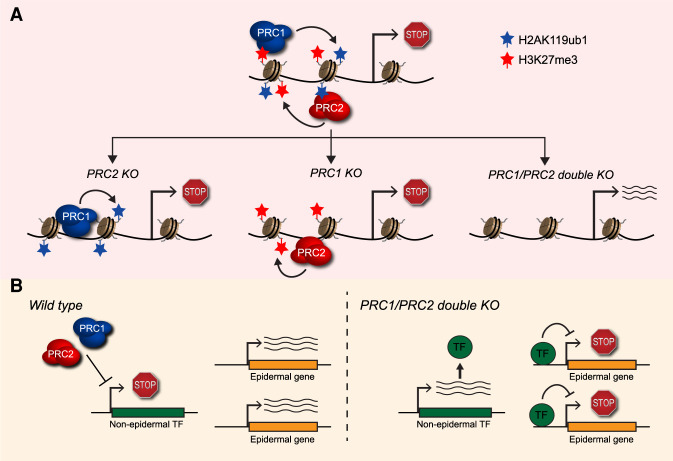
PRC1 and PRC2 can independently repress gene expression yet cooperate to maintain epidermal gene expression patterns and cellular identity. (*A*, *top* panel) Coupling between PRC1 and PRC2 through their capacity to recognize each other's histone modifications is associated with Polycomb-mediated gene repression. However, Cohen et al. (2021) show that at many genes, both PRC1 (*bottom left* panel) and PRC2 (*bottom middle* panel) are able to act independently to partially maintain gene repression. (*Bottom right* panel) Full derepression is only achieved when both PRC1 and PRC2 are ablated. (*B*) In wild-type EpSCs, PRC1 and PRC2 act to maintain the repressed state of nonepidermal transcription factors, thereby protecting the epidermal transcriptional program. However, in the absence of PRC1 and PRC2, misexpression of nonepidermal TFs suppresses the expression of essential epidermal genes.

Cohen et al. (2021) tackle this important point using mouse genetics and the developing epidermis as a model system. Through an elegant series of perturbation experiments using embryonic epidermal progenitor cells, they removed PRC1 and PRC2, individually or in combination, and then examined the resulting effects on skin development. The investigators found that ablation of PRC1 or PRC2 individually had only minor effects on the developing epidermis. However, in contrast to these mild phenotypes, the combined removal of both PRC1 and PRC2 caused catastrophic defects in epidermis differentiation, suggesting that these complexes may have overlapping roles in skin development.

To explore the molecular explanation for these developmental phenotypes, Cohen et al. (2021) then examined gene expression in embryonic epidermal stem cells (EpSCs). In agreement with the observed developmental phenotypes, ablation of PRC1 or PRC2 individually caused a modest derepression of Polycomb target genes (∼1300 genes for PRC1 and ∼500 for PRC2), a proportion of which overlapped. However, when both PRC1 and PRC2 were removed in combination, ∼2500 genes were significantly derepressed. Therefore, if one of the two Polycomb repressive complexes is removed, the remaining complex appears to retain some capacity to repress transcription. Importantly, these findings are consistent with previous work in mouse embryonic stem cell culture in which complete derepression of Polycomb target genes only manifested when both PRC1 and PRC2 were removed (Leeb et al. 2010; King et al. 2018). Together, these observations show that PRC1 and PRC2 must have autonomous yet overlapping functions in repressing target genes despite their biochemical coupling on chromatin (Fig. 1A). Furthermore, it suggests that they can also use independent mechanisms to achieve target gene identification.

The Polycomb repressive system has previously been shown to maintain the silent state of developmental transcription factors in tissues where they should not be expressed (Schuettengruber et al. 2017). It is often suggested that elevated expression of these factors in the absence of Polycomb repression would drive alternative gene expression programs and perturb cell identity (Leeb et al. 2010; Perdigoto et al. 2016). However, evidence directly supporting this in mammals is sparse. Interestingly, within the genes that showed increased expression following removal of both PRC1 and PRC2, Cohen et al. (2021) found ∼300 nonepidermal transcription factors (TFs). Amongst these TFs, the investigators identified 19 that had consensus binding motifs that were enriched at epidermal gene promoters and other putative regulatory elements. Importantly, Cohen et al. (2021) then showed that ectopic expression of different combinations of these TFs in EpSCs was sufficient to suppress the expression of essential epidermal genes. This was consistent with the reduced expression of these genes after PRC1 and PRC2 removal, strongly suggesting that inappropriate expression of nonepidermal transcription factors underpins the developmental phenotypes observed following perturbation of the Polycomb system (Fig. 1B). Interestingly, a recent study examining maintenance of cell fate in the adult mouse intestine also found that disruption of the Polycomb system caused inappropriate expression of a number of normally silenced TFs, and showed that this leads to the re-emergence of an embryonic intestine transcriptional program (Jadhav et al. 2019). Together, these findings provide formal evidence in mammals that the Polycomb system plays an important role in safeguarding cell identity by protecting against inappropriate expression of developmental TFs in cell types where they should not be expressed.

Importantly, the discoveries by Cohen et al. (2021) reinforce at least two emerging themes in Polycomb-dependent gene regulation. First, they highlight that PRC1 and PRC2 must have independent mechanisms to identify target genes that do not intrinsically rely on molecular coupling and recognition of each other's histone modifications. In agreement with this, several distinct PRC1 and PRC2 complexes have recently been shown to include auxiliary proteins with inherent DNA-binding activities that support autonomous binding to Polycomb target genes (Blackledge et al. 2015; Hauri et al. 2016; Laugesen et al. 2019; van Mierlo et al. 2019). Building on this work, in future studies it will be important to further define the molecular logic that enables PRC1 and PRC2 to select their appropriate target genes in specific cell types. Second, Cohen et al. (2021) provide further evidence that PRC1 and PRC2 must have the capacity to repress transcription through distinct mechanisms, despite their molecular coupling on chromatin, as otherwise, removing either complex would yield the same effects on gene expression. A central challenge for future studies remains to understand the detailed molecular mechanisms through which each Polycomb complex counteracts the process of transcription to enable gene repression.

Based on the emerging view that Polycomb complexes have independent yet overlapping roles in repressing target genes, this then raises the important question of what molecular coupling between PRC1 and PRC2 contributes to gene regulation. One possibility is that this additional layer of coupling could enable synergy between the complexes, and as a result provide further robustness in supporting Polycomb-mediated gene repression. This may be particularly important over developmental time scales where gene expression patterns must be maintained to protect cell identity. To examine these possibilities, future work will be required to dissect how development and gene expression are affected when the molecular coupling between PRC1 and PRC2 is disrupted in a manner that does not influence their autonomous functions. Inevitably, the elegant approaches used by Cohen et al. (2021) will be central to answering these fundamental questions about Polycomb system function.

## References

[GAD348257BLAC1] Blackledge NP, Rose NR, Klose RJ. 2015. Targeting Polycomb systems to regulate gene expression: modifications to a complex story. Nat Rev Mol Cell Biol 16: 643–649. 10.1038/nrm4067 26420232 PMC5469428

[GAD348257BLAC2] Cohen I, Bar C, Liu H, Valdes VJ, Zhao D, Galbo PM Jr., Silva JM, Koseki H, Zheng D, Ezhkova E. 2021. Polycomb complexes redundantly maintain epidermal stem cell identity during development. Genes Dev (this issue). 10.1101/gad.345363.120 PMC7919412 33602871

[GAD348257BLAC3] Hauri S, Comoglio F, Seimiya M, Gerstung M, Glatter T, Hansen K, Aebersold R, Paro R, Gstaiger M, Beisel C. 2016. A high-density map for navigating the human Polycomb complexome. Cell Rep 17: 583–595. 10.1016/j.celrep.2016.08.096 27705803

[GAD348257BLAC4] Jadhav U, Cavazza A, Banerjee KK, Xie H, O'Neill NK, Saenz-Vash V, Herbert Z, Madha S, Orkin SH, Zhai H, 2019. Extensive recovery of embryonic enhancer and gene memory stored in hypomethylated enhancer DNA. Mol Cell 74: 542–554.e5. 10.1016/j.molcel.2019.02.024 30905509 PMC6499659

[GAD348257BLAC5] King HW, Fursova NA, Blackledge NP, Klose RJ. 2018. Polycomb repressive complex 1 shapes the nucleosome landscape but not accessibility at target genes. Genome Res 28: 1494–1507. 10.1101/gr.237180.118 30154222 PMC6169895

[GAD348257BLAC6] Laugesen A, Højfeldt JW, Helin K. 2019. Molecular mechanisms directing PRC2 recruitment and H3K27 methylation. Mol Cell 74: 8–18. 10.1016/j.molcel.2019.03.011 30951652 PMC6452890

[GAD348257BLAC7] Leeb M, Pasini D, Novatchkova M, Jaritz M, Helin K, Wutz A. 2010. Polycomb complexes act redundantly to repress genomic repeats and genes. Genes Dev 24: 265–276. 10.1101/gad.544410 20123906 PMC2811828

[GAD348257BLAC8] Perdigoto CN, Dauber KL, Bar C, Tsai PC, Valdes VJ, Cohen I, Santoriello FJ, Zhao D, Zheng D, Hsu YC, 2016. Polycomb-Mediated repression and Sonic Hedgehog signaling interact to regulate Merkel cell specification during skin development. PLoS Genet 12: e1006151. 10.1371/journal.pgen.1006151 27414999 PMC4944976

[GAD348257BLAC9] Schuettengruber B, Bourbon HM, Di Croce L, Cavalli G. 2017. Genome regulation by Polycomb and Trithorax: 70 years and counting. Cell 171: 34–57. 10.1016/j.cell.2017.08.002 28938122

[GAD348257BLAC10] van Mierlo G, Veenstra GJC, Vermeulen M, Marks H. 2019. The complexity of PRC2 subcomplexes. Trends Cell Biol 29: 660–671. 10.1016/j.tcb.2019.05.004 31178244

